# Serum Thyroid Function, Mortality and Disability in Advanced Old Age: The Newcastle 85+ Study

**DOI:** 10.1210/jc.2016-1935

**Published:** 2016-08-23

**Authors:** Simon H. S. Pearce, Salman Razvi, Mohammad E. Yadegarfar, Carmen Martin-Ruiz, Andrew Kingston, Joanna Collerton, Theo J. Visser, Tom B. Kirkwood, Carol Jagger

**Affiliations:** Institute of Genetic Medicine (S.H.S.P., S.R.), Newcastle University, Newcastle upon Tyne NE1 3BZ, United Kingdom; Institute of Health and Society (M.E.Y., C.M.-R., A.K., J.C., T.B.K., C.J.), Newcastle University, Newcastle upon Tyne NE2 4AX, United Kingdom; and Department of Internal Medicine (T.J.V.), Erasmus Medical Center, Rotterdam, The Netherlands

## Abstract

**Context:**

Perturbations in thyroid function are common in older individuals but their significance in the very old is not fully understood.

**Objective:**

This study sought to determine whether thyroid hormone status and variation of thyroid hormones within the reference range correlated with mortality and disability in a cohort of 85-year-olds.

**Design:**

A cohort of 85-year-old individuals were assessed in their own homes (community or institutional care) for health status and thyroid function, and followed for mortality and disability for up to 9 years.

**Setting and Participants:**

Six hundred and forty-three 85-year-olds registered with participating general practices in Newcastle and North Tyneside, United Kingdom.

**Main Outcomes:**

All-cause mortality, cardiovascular mortality, and disability according to thyroid disease status and baseline thyroid hormone parameters (serum TSH, FT_4_, FT_3_, and rT_3_). Models were adjusted for age, sex, education, body mass index, smoking, and disease count.

**Results:**

After adjustment for age and sex, all-cause mortality was associated with baseline serum rT_3_ and FT_3_ (both *P* < .001), but not FT_4_ or TSH. After additional adjustment for potential confounders, only rT_3_ remained significantly associated with mortality (*P* = .001). Baseline serum TSH and rT_3_ predicted future disability trajectories in men and women, respectively.

**Conclusions:**

Our study is reassuring that individuals age 85 y with both subclinical hypothyroidism and subclinical hyperthyroidism do not have a significantly worse survival over 9 years than their euthyroid peers. However, thyroid function tests did predict disability, with higher serum TSH levels predicting better outcomes. These data strengthen the argument for routine use of age-specific thyroid function reference ranges.

## Abstract (precis)

In a cohort of 85 year olds, serum reverse T3 and FT3, but not TSH or FT4, predicted mortality over 9 years. TSH and reverse T3 predicted disability outcomes in men and women, respectively.

Although overt thyroid dysfunction has long been associated with cardiovascular disease, the effects of subtle variations in thyroid function are less clear (1–3). However, this topic has taken on increasing significance, given that it has been recognized that up to 3% of older individuals have a serum TSH concentration that is below the healthy reference interval for younger people (subclinical hyperthyroidism), and approximately 10% have an elevated serum TSH level (subclinical hypothyroidism) (4–7). The high prevalence of these mild or subclinical abnormalities of serum TSH with advancing age has led to a significant body of research concerning the possibility that these changes may presage ill health (2, 8–10). Similarly, the possibility that variations of serum TSH and circulating thyroid hormones even within the healthy reference range may predict current or future disease has also been examined (3, 11).

Twenty years ago, a low serum TSH level was first associated with an increased risk of atrial fibrillation (AF) (12), and this association has been reproduced many times since (13–16). Similarly, longitudinal studies have shown that a low serum TSH concentration is associated with a higher rate of cardiovascular events, osteoporotic fracture, dementia, and death (14, 17–20). However, there is significant heterogeneity within the literature, with some large and well-conducted studies failing to reproduce these associations (13, 21), as well as unresolved questions about causality. Similarly, several studies have found an association of elevated serum TSH with adverse cardiac outcomes, including myocardial infarction (MI), heart failure, and mortality (8, 10, 21–24). Nevertheless, these findings have not been reproduced by all studies (13, 17, 25, 26), suggesting that there may be heterogeneity between the populations studied, or confounding by comorbidities, medications, methodology or other unknown factors. Some authors have suggested that an elevated serum TSH in older age might have no adverse effect on health (7, 27, 28) although one study of individuals age 85 years showed that a high serum TSH at baseline was associated with a favorable outcome compared with that of euthyroid contemporaries (29). However, data from subjects in advanced old age is scarce and this finding has not been confirmed (2). Thus, mild biochemical abnormalities of thyroid function are common with increasing age but their significance remains unclear, particularly in the very old. In this study we examined the relationship between thyroid function and subsequent outcome in a cohort of 85-year-old individuals.

## Participants and Methods

### Participants and study design

Study participants were members of the Newcastle 85+ Study, a longitudinal study of health trajectories and outcomes in a single-year birth cohort (1921) recruited at age 85 years from general (family) practices in Newcastle and North Tyneside, which is described in detail elsewhere (30, 31). The recruited cohort was sociodemographically representative of the general United Kingdom population and included institutionalized older adults (31). At study baseline (2006/07), comprehensive measures of health were collected across multiple clinical, biological, and psychosocial domains. A health assessment—comprising questionnaires, anthropometric measurements, physical and cognitive function tests, and a fasting blood sample—was carried out in the participant's usual residence by a research nurse. General practice medical records were also reviewed to extract data on diagnosed diseases and prescribed medication. In participants who were temporarily hospitalized at the time of recruitment, assessments were undertaken after discharge. All participants provided written informed consent except for those who lacked capacity to consent (for example, in dementia). For the latter group, an opinion was obtained from a close relative or carer, as required by UK law (32). The study was approved by the Newcastle and North Tyneside 1 Research Ethics Committee (Ref: 06/Q0905/2).

## Health assessment, disease counts, and disability

The prevalence of common diseases was determined by evaluation of general practitioner records and/or health assessments. Diseases were scored as either present or absent for 17 selected chronic diseases and a disease count calculated as detailed previously (33). Briefly, these were: hypertension, ischemic heart disease, cerebrovascular disease, peripheral vascular disease, heart failure, atrial flutter or fibrillation, arthritis (osteoarthritis or rheumatoid arthritis), osteoporosis, chronic obstructive pulmonary disease or asthma, other respiratory disease, diabetes mellitus (type 1 or 2), cancer (diagnosed within the previous five years), eye disease, dementia, Parkinson's disease, anemia, and renal impairment (estimated glomerular filtration rate < 30 mL/min/1.73m^2^) [methodology as reported in Collerton et al (31) but omitting thyroid disease from the disease count]. A disability score was created from 17 activities of daily living at baseline, 18 months, 36 months, and 60 months, and, using group-based trajectory modeling that accounted for mortality, four disability trajectories were identified for men and women separately (33). Information on date and cause of death (to ICD-10) were provided from linkage with the national registration system.

## Blood samples and biochemical analysis

After an overnight fast, 40 mL blood was drawn from the antecubital vein between 0700 and 1030 hours. Blood sampling took place between June 2006 and August 2007. Measurement of TSH, FT_4_, and FT_3_ was performed using a Centaur platform (Seimens). Intra-assay coefficients of variation were serum TSH, 3.8% (at 5.3 mU/L); FT_4_, 7.9% (at 13.5 pmol/L) and FT_3_, 3.1% (at 10.5 pmol/L). Serum reverse T_3_ (rT_3_) was assayed in duplicate by RIA, using antiserum 3684 at a final dilution of 1:600 000 and sample volumes of 25 μL, as described (34). Thyroid peroxidase antibodies were assayed by Advia chemiluminescent assay on the Centaur platform with an intra-assay coefficient of variation of 17.7% at a concentration of 87.2 U/L. The following reference ranges were used: TSH, 0.4–4.0 mU/L; FT_4_, 11.0–23.0 pmol/L; FT_3_, 3.5–6.5 pmol/L; TPO antibodies, 0–60 U/L. Overt hypothyroidism was defined as TSH at least 10.00 mU/L or TSH, 4.01–9.99 mU/L and FT_4_ < 10.9 pmol/L; subclinical hypothyroidism was TSH, 4.01–9.99 mU/L and FT_4_ at least 11.0 pmol/L; euthyroid was TSH, 0.40–4.00 mU/L and FT_4_, 11.0–23.0 pmol/L; subclinical hyperthyroidism TSH < 0.399 mU/l, FT_4_ < 23.1, FT_3_ < 6.6; overt hyperthyroidism TSH < 0.099 and either FT_4_ > 23.0 pmol/L or FT_3_ at least 6.6 pmol/L. None of the participants classified as euthyroid from biochemical analysis had thyroid disease recorded in the general practice records.

Both health assessment and general practice records data were available for 845 participants at study baseline, of whom 760 had complete data on serum TSH, FT_3_, FT_4_, and rT_3_. An additional 116 were excluded as they were on thyroid medication or medication interfering with thyroid function (levothyroxine, antithyroid drugs, amiodarone, lithium, corticosteroids). One additional participant was excluded because she was diagnosed with hypopituitarism as part of the study assessments, leaving 643 participants whose mortality outcome was documented until January 1, 2015.

## Statistical analysis

Differences in the baseline characteristics between the groups defined by biochemical status were assessed by χ^2^ tests for the binary variables and Kruskal-Wallis for ordinal and continuous variables (none of which were normally distributed). The time interval for survival was calculated as that between time of blood draw and time of death or January 1, 2015. Kaplan-Meier plots were used to assess the relative risk of mortality by biochemical status separately by sex. Cox proportional hazards models were employed to explore the relative risk of mortality associated with each of the four serum thyroid measures separately (TSH, FT_4_, FT_3_, rT_3_) as continuous variables and for estimating hazard ratios (HRs) and 95% confidence intervals (CIs) adjusted first, for age and sex (Model 1), and then for potential confounders: education (0–9 y, 10–11 y, 12+ y), body mass index (BMI), smoking (none, ex, current) and disease count (Model 2). Separate models were fitted to the entire and the euthyroid cohorts for all-cause mortality and cardiovascular mortality (ICD-10 codes I00-I99). The proportional hazards assumption was tested by examining the Schoenfeld residuals. Potential nonlinear relationships between each of the thyroid measures and all-cause and cardiovascular mortality were examined by fitting restricted cubic splines (35). The effect of the thyroid parameters on disability was examined by fitting ordinal logistic regression models adjusted for the potential confounders listed earlier. Analyses were undertaken in SAS version 9.4 (SAS Institute).

## Sensitivity analyses

To compare our results more directly with the Leiden birth cohort of the same age (29), we repeated the Cox proportional hazards regression modeling adjusting for the same baseline covariates (age, sex, albumin, C-reactive protein [CRP], disease count, mini mental state examination [MMSE] score, disability score, subjective health).

During the first 5 years of the follow-up period, 21 participants were newly treated with levothyroxine and a further participant with antithyroid drugs. We repeated survival analyses excluding these 22 but the results remained the same (data not shown).

## Results

### Mortality by thyroid status

In this population of 643 85-year-olds, 83.1% (n = 534) were euthyroid at baseline, 12.5% (n = 79) had subclinical hypothyroidism, 2.9% (n = 19) had subclinical hyperthyroidism, and less than 1% were overtly hypothyroid (0.9%, n = 6) or hyperthyroid (0.8%, n = 5) (Table 1). Table 1 also shows the baseline characteristics for all participants (n = 643) and separately for those classified as euthyroid (n = 534). At baseline, biochemical thyroid status (including euthyroid, subclinical, and overt hypo- and hyper-thyroidism) was associated with years in education (χ^2^ = 10.86; df = 4; *P* = .028), medical history of ischemic heart disease (χ^2^ = 9.27; df = 2; *P* < .001), serum albumin (F = 4.33; df = 2; *P* = .014), CRP (F = 5.06; df = 2; *P* = .007) and disability score (χ^2^ = 7.29; df = 2; *P* = .03) (Supplemental Table 1).

**Table 1.** Baseline Characteristics of Study Sample

Characteristic	Total (n = 643)	Euthyroid (n = 534)
Age, y, mean (sd)	85.5 (0.4)	85.5 (0.4)
Male, % (n)	42.0 (270)	43.6 (233)
BMI, mean (sd)	24.4 (4.4)	24.5 (4.5)
Current smoker, % (n)	5.6 (36)	5.3 (28)
Basic education, % (n)	63.6 (404)	64.2 (339)
Living in care home, % (n)	8.7 (56)	8.6 (46)
Medical history, % (n)		
Diabetes	14.1 (91)	14.2 (76)
Hypertension	58.2 (374)	59.0 (315)
IHD	34.4 (221)	37.3 (196)
Stroke	13.8 (89)	13.7 (73)
Cancer	6.4 (41)	6.2 (33)
Disease count, median (IQR)	4 (3–6)	4 (3–6)
Disability score, median (IQR)	3 (1–7)	3 (1–7)
MMSE score, median (IQR)	28 (25–29)	28 (25–29)
Fair or poor self-rated health, % (n)	19.9 (120)	20.9 (110)
Survival time, mo, median (IQR)	65.6 (32.6–92.6)	64.6 (32.4–91.1)
Deaths, % (n)	68.4 (440)	70.2 (375)
Cardiovascular deaths, % (n)	39.2 (252)	40.6 (217)
TSH^a^, mU/L	1.87 (0.27–6.53)	1.73 (0.58–3.80)
FT_3_^a^, pmol/L	4.54 (3.60–5.5)	4.52 (3.60–5.40)
FT_4_^a^, pmol/L	15.20 (11.0–21.0)	15.24 (11.0–20.0)
rT_3_^a^, nmol/L	0.40 (0.25–0.67)	0.40 (0.26–0.67)
TPOAbs positive, % (n)	15.9 (102)	12.6 (67)
Albumin^a^, g/L	40.03 (33.00–46.00)	40.03 (33.00–46.00)
C reactive protein^a^, mg/L	2.65 (0.30–52.20)	2.67 (0.30–52.20)
Overt hypothyroid	0.9 (6)	—
Subclinical hypothyroid	12.3 (79)	—
Euthyroid	83.1 (534)	534 (100)
Subclinical hyperthyroid	2.9 (19)	—
Overt hyperthyroid	0.8 (5)	—

Abbreviations: IHD, ischemic heart disease; IQR, interquartile range; TPOAbs, thyroid peroxidase antibodies.

a Geometric mean, 2.5–97.5th percentiles

**Figure 1.**
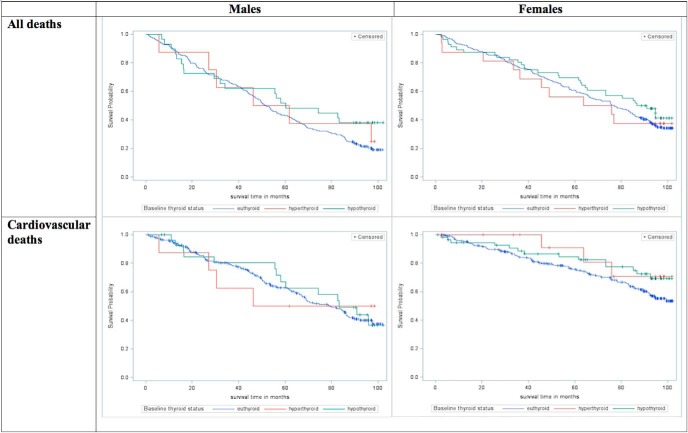
Cumulative survival (all deaths and cardiovascular deaths) of participants based on thyroid status, by sex.

Owing to the small number of overt hyper- and hypothyroid subjects, we combined the subclinical and overt biochemical categories for plotting the Kaplan-Meier curve for all-cause and cardiovascular mortality (Figure 1). No significant difference was found between the categories for all-cause mortality (male: *P* = .32; female: *P* = .54) or cardiovascular mortality (male: *P* = .94; female: *P* = .18). Nor was association found between thyroid status and either all-cause mortality or cardiovascular mortality after adjustment for age and sex, or after further adjustment for potential confounding factors (Supplemental Table 2).

### Individual serum thyroid parameters

Analysis of each of the serum thyroid hormones individually showed that all-cause mortality was negatively associated with FT_3_ concentrations (HR = 0.81; 95% CI, 0.73–0.91; *P* < .001) and positively associated with levels of rT_3_ (HR = 1.23; 95% CI, 1.12–1.34; *P* < .001) although only rT_3_ remained significantly associated after adjustment for the potential confounding baseline factors of education, BMI, smoking status, and disease count (Table 2). Similar trends were found for cardiovascular mortality although, due to smaller number of deaths, the association of rT_3_ with cardiovascular mortality was no longer significant after adjustment for baseline factors (Table 2). When analyses were repeated for the subset of euthyroid participants (n = 534), the same associations were found although all-cause mortality associations for both FT_3_ and rT_3_ remained significant after adjustment for baseline factors (Table 2).

**Table 2.** Relationship Between Thyroid Hormones and All-cause and Cardiovascular Mortality in the Full Cohort and in Euthyroid Participants; HRs (per Unit Increase) and 95% CIs

	Model 1^a^			Model 2^b^		
	HR^c^	95% CI	*P*-Value	HR^c^	95% CI	*P*-Value
Full cohort (n = 643)						
All-cause mortality						
TSH	0.92	0.78–1.09	.32	0.87	0.71–1.07	.19
FT_3_	0.81	0.73–0.91	<.001	0.91	0.81–1.01	.08
FT_4_	1.09	0.99–1.21	.08	1.07	0.96–1.19	.22
rT_3_	1.23	1.12–1.34	<.001	1.18	1.07–1.31	.001
Cardiovascular mortality						
TSH	1.03	0.88–1.19	.74	0.96	0.74–1.25	.76
FT_3_	0.85	0.74–0.97	.02	0.97	0.84–1.12	.70
FT_4_	1.03	0.90–1.17	.71	0.99	0.86–1.15	.93
rT_3_	1.22	1.08–1.38	.002	1.13	0.99–1.29	.07
Euthyroid at baseline (n = 534)						
All-cause mortality						
TSH	0.64	0.43–0.95	.027	0.72	0.47–1.09	.12
FT_3_	0.75	0.66–0.84	<.001	0.84	0.74–0.96	.008
FT_4_	1.05	0.94–1.18	.40	1.03	0.90–1.17	.69
rT_3_	1.22	1.11–1.35	<.001	1.17	1.05–1.31	.006
Cardiovascular mortality						
TSH	0.74	0.44–1.24	.26	0.88	0.51–1.52	.65
FT_3_	0.78	0.66–0.91	.002	0.90	0.77–1.06	.22
FT_4_	1.00	0.86–1.17	.98	0.96	0.81–1.13	.61
rT_3_	1.21	1.06–1.38	.006	1.10	0.95–1.28	.20

a Adjusted for age and sex

b Adjusted for age, sex, education, BMI, smoking, disease count

c HR per sd increase of: 3.223 mU/L TSH, 0.496 pmol/L FT_3_, 2.470 pmol/L FT_4_, 0.109 nmol/L rT_3_.

**Figure 2.**
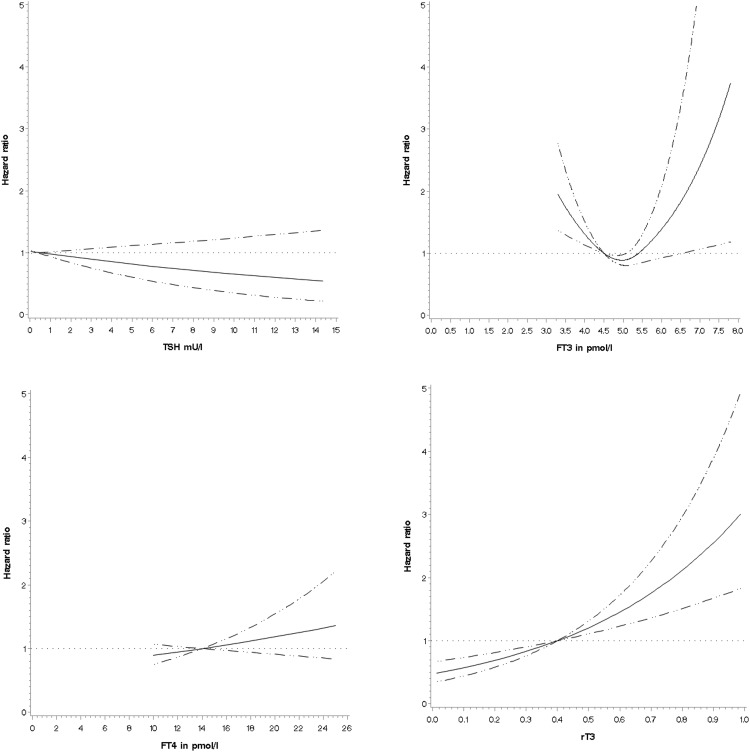
Restricted cubic spline curves of dose-response relationship between TSH, FT_3_, FT_4_, rT_3_, and all-cause mortality, adjusted for age, sex, education, smoking status, BMI, and disease count, in the Newcastle 85+ Study.

Test of the proportional hazards assumptions showed that none of the variables violated the assumptions. Analysis of the individual thyroid hormones by restricted cubic splines to check for nonlinear associations with all-cause and cardiovascular mortality in the entire cohort showed that only FT_3_ had a nonlinear association with mortality, after adjustment for potential confounders, suggesting that both low and high FT_3_ (> 6.75 pmol/L) values were associated with increased all-cause mortality (Figure 2 and Supplemental Figure 1).

To examine whether our results were dependent upon the choice of confounders, we repeated analyses using the same confounding factors as Gussekloo et al (29), (age, sex, albumin, CRP, disease count, MMSE score, disability score, subjective health) and restricting survival to 4 years. Results were unchanged with only rT_3_ showing a significant relationship with mortality.

### Disability

We examined the relationship between each of the thyroid function measures (TSH, FT_3_, FT_4_, rT_3_) and disability trajectory during the first 60 months of the study, for men and women separately (Table 3). The distributions of the thyroid hormones for each disability trajectory are shown in Supplemental Table 3. For women there was a significant trend toward increasing disability with decreasing levels of TSH, FT_3_, and increasing levels of rT_3_, although after adjustment for potential confounders only rT_3_ remained significantly associated with increased disability (Table 3). Trends for men were in a similar direction to women although after adjustment, only decreasing levels of TSH were predictive of increased disability. FT_4_ was not associated with disability trajectory for either men or women.

**Table 3.** Relationship Between Thyroid Hormones and Male and Female Disability Trajectories; Odds Ratios (per sd Increase) and 95% CIs

	Model^a^			Model 2^b^		
	OR^c^	95% CI	*P*-Value	OR^c^	95% CI	*P*-Value
Male disability trajectories						
TSH	1.03	0.88–1.20	.72	0.62	0.40–0.97	.038
FT_3_	0.68	0.54–0.85	<.001	0.80	0.63–1.03	.08
FT_4_	1.14	0.91–1.44	.26	1.16	0.89–1.50	.26
rT_3_	1.17	0.95–1.45	.13	1.10	0.88–1.39	.40
Female disability trajectories						
TSH	0.58	0.40–0.85	.005	0.74	0.48–1.14	.17
FT_3_	0.77	0.63–0.94	.009	0.92	0.73–1.15	.44
FT_4_	1.10	0.90–1.34	.37	1.14	0.90–1.44	.29
rT_3_	1.44	1.18–1.76	<.001	1.28	1.01–1.62	.04

Abbreviation: OR, odds ratio.

a Adjusted for age

b Adjusted for age, education, BMI, smoking, disease count less thyroid disease

c ORs per sd increase of: 3.223 mU/L TSH, 0.496 pmol/L FT_3_, 2.470 pmol/L FT_4_, 0.109 nmol/L rT_3_.

## Discussion

### Mortality

The significance of mild disturbances of thyroid function in older individuals is poorly understood, particularly in the very old. The most frequent abnormality found is a raised serum TSH with circulating thyroid hormone concentrations that are within reference range, correlating with the state of subclinical hypothyroidism, and some studies suggest an adverse effect of this state (8, 10, 22, 24) whereas others show a neutral or even protective effect on outcome (2, 17, 25, 29, 36, 37). Notably, the Leiden 85+ study, a cohort study of 85-year-olds with a similar design to our study, found that subclinical hypothyroidism was associated with reduced all-cause mortality and cardiovascular mortality (29). This finding, along with the results of familial studies of the oldest old (7, 38), suggests that an elevated serum TSH may be positively associated with longevity and favorable outcomes in the very old. This led us and others to suggest that there may be age-related differences in the vascular risk associated with a raised serum TSH, with higher risks found in younger individuals that attenuated with advancing age (27, 39, 40). In the current study we have examined a substantial cohort of 85-year-olds and followed their outcome for up to 9 years. We find that baseline serum TSH had no substantial association with all-cause or vascular mortality in our cohort, which confirms the findings of many previous studies (2, 13, 17, 25, 29, 36), albeit in a cohort of older participants. These findings, therefore, provide further reassurance that subclinical hypothyroidism is a benign state in advanced older age (2, 29). Furthermore, we also show that variation of serum TSH within the reference interval is not associated with impaired survival. Indeed, perhaps parallel to the findings of the Leiden 85+study (29), when only euthyroid participants were considered there is a hint of a protective effect of higher serum TSH on all-cause mortality, although this did not persist after adjustment for comorbidities. In addition, lower serum TSH was associated with disability in men, with a nonsignificant though similar trend in women. Whether there could be a survival benefit associated with subclinical hypothyroidism at this age remains an open question, and our study does not exclude this possibility. However, sensitivity analysis in our cohort, using exactly the same adjustment factors as the Leiden 85+ study failed to reproduce this finding in participants of the same age, albeit in a later birth cohort.

In contrast with subclinical hypothyroidism, many previous studies have shown that low serum TSH is associated with poor outcomes, either increased all-cause or vascular mortality, or AF (12–14, 17, 29, 36, 37). This has led to suggestions that patients with low TSH should be aggressively treated for hyperthyroidism to avoid these complications despite a complete lack of compelling (level 1) evidence to support the effectiveness of this strategy (1, 41, 42). Although this current study does not support an increase in mortality associated with low TSH, there are a relatively low number of participants with subclinical or overt hyperthyroidism in this study (24 in total), so this result is less robust than that for individuals with higher serum TSH concentrations or subclinical hypothyroidism. Nevertheless, the finding of no adverse effect associated with lower serum TSH concentration within the reference range is consistent with several other studies, and broadly reassuring.

Serum T_4_ is metabolized either to the active thyroid hormone T_3_ or to the inactive rT_3_ in a reciprocal manner depending upon the relative actions of the tissue deiodinase enzymes (deiodinase types 1 to 3). Reduced circulating concentration of free T_3_ is a sensitive marker of ill health (43). Indeed, fasting, cold exposure, and even minor infective or inflammatory disease are sufficient to reduce serum FT_3_ and elevate rT_3_ in otherwise-healthy individuals (44, 45). In our analysis, higher serum rT_3_ was associated with all-cause mortality in the fully adjusted model: that is independent of prevalent disease burden. In contrast, lower FT_3_ was also associated with both all-cause and cardiovascular mortality but following full correction for disease burden, these associations were lost. These findings broadly confirm those of previous studied cohorts of older people (2, 29), and are in line with the known pathophysiological mechanisms whereby low FT_3_ and higher rT_3_ are nonspecifically associated with increasing ill health and disease burden (44, 45). Interestingly, our analysis is one of only a few to look at serum rT_3_ in individuals of this age, and our data suggest that high rT_3_ is a stronger predictor of all-cause mortality than low FT_3_, independent of disease burden. This finding is consistent with a longitudinal study of 403 Dutch males age 73–94 years in which a group with higher rT_3_ but normal FT_3_ had better performance status than those with lower FT_3_ (43). One explanation may be that rT_3_ is more sensitive to the presence of preclinical morbidities (eg, early tissue inflammation or covert cancer); the alternative being that our disease count incorporating 17 common conditions is incomplete and that serum rT_3_ is sensitive to uncounted conditions not included in the adjustment. Our deeper examination of the relationship between thyroid hormones and mortality by fitting cubic splines showed that most of the hormones were linearly related to mortality. The exception was FT_3_ where both low and high values were predictive of mortality in the very old.

### Disabilities

Scant data exist about the relationship between thyroid function, functional status, and future disability in this age group (43, 46, 47). Our analysis showed that baseline serum TSH, FT_3_, and rT_3_ each predicted future disability with the strongest effects seen in women. However, given that these associations are attenuated following adjustment by baseline disease count and BMI, these factors may be on the causal pathway between thyroid function and disability. The association of disability with lower TSH in men is particularly interesting given that, in cohort studies, subclinical hyperthyroidism or lower serum TSH have been associated with many involutional disease states including dementia, osteoporosis, AF, muscle weakness, low bone density, and fracture (12–14, 18, 20, 48, 49). It has widely been assumed that these associations are caused by subclinical hyperthyroidism with detrimental effects of excess thyroid hormone, for instance its proarrhythmic effect on the heart leading to AF, despite the fact that serum thyroid hormones are within reference range (1, 13–15, 29, 41, 42). Our findings that lower serum TSH predicts future disability, even when adjusted for baseline disease count, suggests that in advanced old age, lower TSH (and in the unadjusted analysis, lower FT_3_/higher rT_3_) correlates with an increasing burden of nonthyroidal disease. This is consistent with the Health ABC study, showing that in a cohort with a mean age of 75 years, the group with higher serum TSH (4.5–6.99 mU/L) had faster gait speed and better cardiorespiratory fitness than those with lower TSH (46).

### Limitations

Our prospective observational study has some limitations. A relatively low number of individuals were studied and so with lower numbers of vascular events, there was less power to detect a significant difference in cardiovascular deaths, compared with all-cause mortality. However, this is still the largest study of this kind in this age group, as far as we are aware. In addition, we assessed 17 common comorbid conditions for which we adjusted in our multivariate analyses. It is possible that there may be other less common yet influential conditions that have not been taken into account. Furthermore, similar to most other longitudinal observational cohort studies, there may be a bias toward healthier people participating. In particular, it seems probable that individuals with sensory impairments may have been under-represented in the cohort. Nevertheless, in contrast with most studies of this age group, we did include participants with advanced frailty living in supervised care and nursing homes as well as those with dementia, so our results should be representative for age-matched populations. Thus, these self-evident potential limitations, as well as other unknown confounders, may also have influenced the results of our study.

### Summary

In a cohort of 85-year-olds, baseline serum rT_3_ was associated with all-cause mortality during a 9-year period of followup, and may be a more sensitive marker for nonthyroidal illness than FT_3_. No significant mortality associations were found with serum TSH, FT_3_, or FT_4_. However, in men lower serum TSH, and in women higher rT_3_ predicted disability. Although it is well established that low FT_3_ is associated with nonthyroidal illness, our findings are consistent with reduced serum TSH also being a marker for disability in older age. These data suggest that there is little detriment associated with subclinical hypothyroidism in this age group.
